# New Appliances and Things Medical

**Published:** 1894-09-15

**Authors:** 


### NEW APPLIANCES AND THINGS MEDICAL.

[All preparations, appliances, novelties, &c., of which a notice is desired, should be sent for the Editor, to care of The Manager 428, Strand, London, W.C.]

### THE SCOTTISH FLUID BEEF COMPANY (LIMITED), Edinburgh.

This company has sent us specimens of their speciality, "Vimbos." This is a fluid extract of beef containing a very large percentage of the albuminous elements of the meat. It is now well understood that mere extract of meat, however good, is only a stimulating adjunct to other foods, and not a food in itself—it largely fails in its usefulness. "Vimbos," however, is not only a stimulating adjunct, but possesses good food value in addition, and is so, in fact, a most important form of concentrated nourishment, the value of which cannot be over-estimated in those cases of treatment where the feeding of the patient is all important. "Vimbos" is an excellent specimen of the modern methods of preparing meat in a fluid form. It can be used either diluted with water as soup, or as it is supplied without admixture by spreading it on bread, and can so be eaten with a relish by the most fastidious of invalids.

